# Integrated Analyses of Gut Microbiome and Host Metabolome in Children With Henoch-Schönlein Purpura

**DOI:** 10.3389/fcimb.2021.796410

**Published:** 2022-01-25

**Authors:** Min Wen, Xiqiang Dang, Shipin Feng, Qingnan He, Xiaoyan Li, Taohua Liu, Xiaojie He

**Affiliations:** ^1^ Department of Pediatrics, The Second Xiangya Hospital, Central South University, Changsha, China; ^2^ Laboratory of Pediatric Nephrology, Institute of Pediatrics, The Second Xiangya Hospital, Central South University, Changsha, China; ^3^ Department of Pediatrics, The Third Xiangya Hospital, Central South University, Changsha, China; ^4^ Department of Pediatric Nephrology, Chengdu Women’s and Children’s Central Hospital, School of Medicine, University of Electronic Science and Technology of China, Chengdu, China

**Keywords:** gut microbiota, host metabolome, Henoch-Schönlein purpura, unsaturated fatty acid, Arachidonic acid, 16S rDNA

## Abstract

Recent studies have shown that intestinal microbes and metabolites are involved in the pathogenesis of many diseases. However, whether and how they are related to Henoch–Schönlein purpura (HSP) has yet to be understood. This work is designed to detect gut microbes, intestinal and serum metabolites in children with HSP, trying to discover the etiology and pathogenesis of HSP. A total of 86 children were recruited in this study, namely, 58 children with HSP (HSP group) and 28 healthy children as control groups (CON group). 16S rDNA amplicon sequencing technology and UPLC-QTOF/MS non-targeted metabolomics analysis were used to detect the intestinal microbes and metabolites, and also multi-reaction monitoring technology for detecting serum arachidonic acid (AA) and its metabolites. Then, correlation analysis was performed to explore the possible interaction between the differential gut microbes and metabolites. As a result, at the microbiota family level, the CON group had an advantage of *Coriobacteriaceae* while the HSP group had a dominant *Bacteroidaceae*. Five kinds of bacteria in the HSP group were significantly enriched at the genus level, and seven kinds of bacteria were significantly enriched in the CON group. A total of 59 kinds of gut metabolites significantly differ between the two groups, in which most are lipids and peptides. Spearman correlation analysis showed that *Bacteroides*, *Dialister*, and *Agathobacter* were associated with unsaturated fatty acids, especially AA metabolism. Then, we tested the AA related metabolites in serum and found thromboxane B2, leukotriene B4, prostaglandin D2, 9S-hydroxyoctadecadienoic acid, and 13S-hydroxyoctadecadienoic acid significantly changed. In conclusion, children with HSP had dominant *Bacteroidaceae* and decreased *Coriobacteriaceae* in the family level of gut microbes, and also lipids and peptides changed most in the gut metabolites. Our data suggested that the biosynthesis and metabolism of unsaturated fatty acids, especially AA and its metabolites, might participate in the occurrence and development of HSP.

## 1 Introduction

Gut microbiota is a complex ecosystem, namely, bacteria, fungi, viruses, etc., of which bacteria account for more than 90% (Zhong et al., 2018). Intestinal microbes and the human immune system symbiotically interact with each other. In recent years, with the development of intestinal microbial 16S rDNA and metagenomic sequencing technology, large researchers have found that the occurrence of related diseases such as systemic lupus erythematosus, rheumatoid arthritis, and type 1 diabetes are closely related to the change of intestinal microecology (Neuman and Koren, 2017; Ruiz et al., 2018; Wang et al., 2018; Zhong et al., 2018). At the same time, their main intestinal metabolites such as arachidonic acid (AA), short-chain fatty acids (SCFAs), and amino acids, lipids were detected to affect intestinal epithelial cells, even the immune homeostasis, material metabolism, and central nervous system (Chen et al., 2012; Johnson et al., 2015; Liu et al., 2017; Mizuno et al., 2017; Zhong et al., 2018). As a major component of cell membrane lipids, AA is mainly metabolized by three kinds of enzymes: cyclooxygenase (COX), lipoxygenase (LOX), and cytochrome P450 (CYP450) enzymes (Martin et al., 2016; Bavaresco et al., 2020). Based on these three metabolic pathways, AA could be converted into various metabolites, that trigger different inflammatory responses. For example, an increased level of prostaglandin (PG), thromboxane A2 (TXA2), and leukotriene B4 (LTB4) result in inflammatory damage to the kidney (Wang et al., 2019).

Henoch–Schonlein purpura (HSP), also called IgA vasculitis (Ozen and Sag, 2020), is an IgA immune complex-mediated vasculitis, mainly common in children between 2 and 8 years old, with a high prevalence rate and unclear pathogenesis (Jennette et al., 2013). The clinical manifestation of HSP is a characteristic hemorrhagic rash of the limbs, especially the symmetry of the lower limbs, which may be accompanied by multi-system organ involvement such as the digestive tract, joints, and kidneys (Ruperto et al., 2010). What is currently considered is that HSP may be related to the immune damage mediated by IgA-1 (Novak et al., 2007; Hetland et al., 2017). Serum galactose deficient IgA l levels are increased, and the combination of soluble IgA FCα receptors to form immune complexes deposited in the kidney may be an important mechanism leading to purpuric nephritis (Chen and Mao, 2015; Suzuki et al., 2018). In addition, fatty acid metabolites produced by gut microbiota such as AA and its metabolites are involved in the inflammatory processes, and neutrophil infiltration is even an important mediator in the process of vascular injury in HSP (Chen et al., 2008; Fu et al., 2018). The humoral and cellular immunity related to the above-mentioned mechanisms may be related to immune disorders caused by changes in intestinal microbes and their metabolites. So, research on the mechanism of HSP in the direction of intestinal microbial metabolites deserves a deeper study.

The purpose of our project was to detect intestinal microbes and metabolites in children with HSP, exploring the potential role of gut microbiota and metabolites, especially AA, playing on the HSP by comparing to the healthy group.

## 2 Materials and Methods

### 2.1 Sample Collection

A total of 58 patients younger than 18 years old with HSP (28 males,30 females) were recruited for this study, all of whom were inpatients or outpatients of the Second Xiangya Hospital of Central South University between December 2018 and July 2019. All HSP patients (HSP group) met the HSP diagnostic criteria recognized by the American Rheumatology Association and the EULAR/PreS (Deng et al., 2010), which is palpable purpuric rashes in the extremities, particularly the lower extremities (necessary) with any of the following: 1) diffuse abdominal pain; 2) biopsy showing significant IgA deposition; 3) acute arthritis or joint pain in any joint; and 4) manifestations of kidney damage [hematuria and (or) proteinuria]. The healthy control group (CON group) consisted of 28 healthy children who were in good health with no history of liver and gastrointestinal diseases, diabetes, hypertension, or autoimmune diseases, according to the medical history investigation and relevant physical examination results from the Health Examination, confirmed by screening questionnaires. Ethical approval for this study was obtained from the Ethics Committee of the Second Xiangya Hospital of Central South University (No XXJ2018-11). All the written informed consent was signed by parents before the study.

Fresh stool samples of each subject were collected in 2 tubes, >400 mg each, frozen immediately, and stored at −80°C until they were processed. Heparin anticoagulated tubes were used to collect approximately 3 ml whole blood samples from all study objects in the early morning after overnight fasting. These were centrifuged at 1,300–2000*g* at 4° C for 10 min and upper plasma were taken (not less than 0.3 ml), which were quickly frozen in liquid nitrogen before storing at −80°C to wait for testing.

### 2.2 16S rDNA Microbiota Analysis

#### 2.2.1 Sequencing

Total genomic DNA from samples was extracted using the CTAB/SDS method (Hu et al., 2021). DNA concentration and purity were monitored on 1% agarose gels. According to the concentration, DNA was diluted to 1 ng/μl using sterile water. The primers were: 16S V4-V5: 515F-907R. 16S rRNA genes were amplified using specific primers with barcodes. All PCRs were carried out in 30 μl reactions with 15 μl of Phusion^®^ High-Fidelity PCR Master Mix (New England Biolabs), 0.2 μM forward and reverse primers, and approximately 10 ng of template DNA. Thermal cycling consisted of initial denaturation at 98°C for 1 min, followed by 30 cycles of denaturation at 98°C for 10 s, annealing at 50°C for 30 s, and elongation at 72°C for 60 s. Finally, 72°C for 5 min. The same volume of 1× loading buffer (containing SYBR green) was mixed with the PCR products, and electrophoresis was performed on a 2% agarose gel for detection. Samples with bright main bands between 400 and 450 bp were chosen for further experiments. PCR products were mixed in equidensity ratios. Then, mixed PCR products were purified with a GeneJET Gel Extraction Kit (Thermo Scientific). Sequencing libraries were generated using the NEB Next^®^Ultra™ DNA Library Prep Kit for Illumina (NEB, USA) following the manufacturer’s recommendations, and index codes were added. The library quality was assessed on the Qubit@ 2.0 Fluorometer (Thermo Scientific) and Agilent Bioanalyzer 2100 system. Finally, the library was sequenced on an Illumina MiSeq platform, and 250 bp/300 bp paired-end reads were generated.

#### 2.2.2 Data Analysis

Sequence analysis was performed by the UPARSE software package using the UPARSE-OTU and UPARSE-OTUref algorithms. In-house Perl scripts were used to analyze the alpha (within samples) diversity and beta (among samples) diversity. Sequences with ≥97% similarity were assigned to the same OTUs (operational taxonomic units). We selected a representative sequence for each OTU and used the RDP classifier to annotate taxonomic information for each representative sequence. To compute the alpha diversity, we verified the OTU table with calculated three metrics: Chao estimates the species abundance; observed species estimates the number of unique OTUs found in each sample and the Shannon index. Cluster analysis was preceded by principal component analysis (PCA) using the QIIME software package. We used unweighted UniFrac distance for principal coordinate analysis (PCoA) and unweighted pair group method with arithmetic mean clustering.

To confirm differences in the abundances of individual taxa between the two groups, Metastats software was utilized. LDA Effect Size (LEfSe) analysis was used for the quantitative analysis of biomarkers within different groups. ANOSIM and MRPP (multiresponse permutation procedure) were performed based on Bray–Curtis dissimilarity distance matrices to identify differences in microbial communities between the two groups.

### 2.3 Metabolomics

#### 2.3.1 GC–MS Analysis

A total of 58 samples of HSP patients and 28 samples of the control group were mixed in equal quantities to prepare Quality Control (QC) samples. Samples (60 mg) were mixed with 200 μg water and vortexed for 60 s before being added to 800 µl methanol acetonitrile (Merck, 1499230-935) solution (1:1, v/v), followed by vortexing for 60 s and low-temperature ultrasound for 30 min twice. Stool samples were stored at −20°C for 1 h to precipitate protein. Finally, the samples were centrifuged for 20 min at 14,000 RCF at 4°C, and the supernatant liquor was collected for freeze-drying and stored at −80°C.

For GC analysis, the samples were separated by an Agilent 1290 Infinity LC ultra-high-performance liquid chromatography (UHPLC) HILIC column at 25°C with a flow rate of 0.3 ml/min. The mobile phase was the composite of A (water, 25 mM ammonium acetate, 25 mM ammonia) and B (acetonitrile). Gradient elution procedures were as follows: B was 95% in 0–1 min, then changed linearly from 95 to 65% in 1–14 min and 65 to 40% in 14–16 min, and maintained at 40% in 16–18 min before changing linearly from 40 to 95% in 18–18.1 min, maintaining at 95% in 18.1–23 min. The samples were placed in an automatic sampler at 4°C throughout the analysis. To avoid the influence caused by the signal fluctuation of the instrument, a random sequence was used for the continuous analysis. QC samples were inserted into the sample queue for monitoring and evaluation of the system.

For Q-TOF MS analysis, electrospray ionized positive ions, and negative ions were detected. The samples were separated by UHPLC and analyzed by a Triple TOF 5600 mass spectrometer (AB SCIEX). The ESI source conditions after HILIC chromatographic separation were as follows: Ion Source Gas 1 (Gas1): 60, Ion Source Gas 2 (Gas2): 60, Curtain gas (CUR): 30, source temperature: 600°C, Ion Spray Voltage Floating (ISVF) ± 5,500 V (positive and negative modes); TOF MS scan m/z range: 60–1,000 Da, product ion scan m/z range: 25–1,000 Da, TOF MS scan accumulation time 0.20 s/spectra, product ion scan accumulation time 0.05 s/spectra. The second-order mass spectrum was obtained by information-dependent acquisition (IDA) with a high sensitivity mode, a declustering potential (DP): of ±60 V (positive and negative modes), collision energy of 35 ± 15 eV, and IDA excluded isotopes within 4 Da with 6 candidate ions to monitor per cycle (Wen et al., 2021).

#### 2.3.2 MRM Analysis

Multi-reaction monitoring technology (MRM) uses standard products as a reference to detect and analyze specific AA metabolite groups in a targeted and specific manner (Meng et al., 2021), and obtain absolute quantitative results of target metabolites, with strong specificity and high sensitivity, features of high accuracy. A total of 14 kinds of AA related metabolite groups include thromboxane B2 (TXB2), LTB4, leukotriene D4 (LTD4), PGE2, PGD2, PGF2α, 8-iso-PGF2α, 6-keto-PGF1α, 9S-hydroxyoctadecadienoic acid (9S-HODE), 13S-HODE, 12S-hydroxyeicosatrienoic acid (12S-HETE), 15S-HETE, 14 (Hetland et al., 2017)-EpETE, and docosahexaenoic acid (DHA).

Slowly thaw the sample at 4°C, add 500 μl of 1% FA methanol (including BHT), vortex to mix, sonicate in an ice bath for 30 min, and centrifuge at 14,000*g* at 4°C for 10 min, and take the equivalent of 40 mg of supernatant to be loaded. The HLB μelution system extracts arachidonic acids, activates with 200 μl methanol, equilibrates with 200 μl water, loads the sample, washes with 200 μl water, washes with 200 μl 10% methanol solution, and washes with 50 μl acetonitrile aqueous solution (acetonitrile: water: formic acid) = 80:20:0.02; v/v/v). Multiquant software was used to extract the chromatographic peak area and retention time. The retention time was corrected with the standard substance of arachidonic acid, and the metabolite identification was carried out. The original data were converted into the mzXML format by ProteoWizard, and then the XCMS program was used for peak alignment, retention time correction, and peak area extraction.

#### 2.3.3 Data Analysis

Accurate mass number matching (<25 PPM) and secondary spectrogram matching were used to identify the metabolite structure, and the laboratory database was retrieved. After pretreatment by Pareto scaling, multidimensional statistical analysis was performed, namely, principal component analysis (PCA), supervised partial least square discriminant analysis (PLS-DA), and orthogonal partial least square discriminant analysis (OPLS-DA). One-dimensional statistical analysis included the Student’s t-test and multivariate analysis of variance. In this study, Variable important in projection (VIP) >1 was selected and the metabolites with p <0.05 were analyzed by univariate statistical analysis.

### 2.4 Association Analysis

Principal component analysis (PCA) was performed with SIMCA Version 14.1 using quantitative data from the two omics analyses. All differential expressed microbes and metabolites were queried and mapped to pathways based on the online Kyoto Encyclopedia of Genes and Genomes (KEGG, http://www.kegg.jp/). Enrichment analysis was also performed. R Version 3.5.1 was used to combine the KEGG annotation and enrichment results of the two omics approaches. A Venn diagram and a bar plot were drawn. Spearman correlation analysis coefficients among the differential microbes and metabolites were loaded into Cytoscape Version 3.5.1, and the correlation network was calculated. Then draw a scatter plot for each association result.

## 3 Results

### 3.1 Clinical Data of the Recruited Children

A total of 58 patients younger than 18 years old with HSP (28 males,30 females) were recruited for this study. The mean age of the HSP group was 9.17 ± 3.08 years, and that of the CON group was 10.60 ± 3.66 years, showing no significant difference (p >0.05). The main clinical symptoms in patients with HSP included palpable rashes in the extremities (48.28%), arthritis (20.70%), gastrointestinal symptoms (20.70%), and renal damage (37.93%). HSP patients were divided into three groups according to the drug-using history, namely, the untreated group (UG, diagnosed as HSP for the first time and has not been treated with any immunotherapy drugs, 25.86%), the regular treated group (RG, used glucocorticoids with or without immunotherapy drugs of tacrolimus, cyclosporine, cyclophosphamide, 51.72%), and the withdrawal group (WG, completely discontinued with any drugs for at least 3 months and did not show clinical symptoms, 22.41%). The glucocorticoids treatment was oral prednisone at 0.5–1 mg/kg/d (maximum 60 mg), then gradually reduced the dose after one week or intravenous methylprednisolone at 1–1.5 mg/kg/d. Simultaneously, the renal damage (RD) group (37.93%) and no renal damage (NO-RD) group (62.07%) were divided according to the renal damage, which is defined as gross hematuria or microscopic hematuria, and proteinuria meeting any of the following: 1) Routine urine protein is positive three times in a week, 2) the 24-hour urine protein quantitative is greater than 150 mg, and 3) The urine microalbumin was higher than normal three times in a week.

### 3.2 Intestinal Dysbiosis in HSP Children

**Figure 1:**
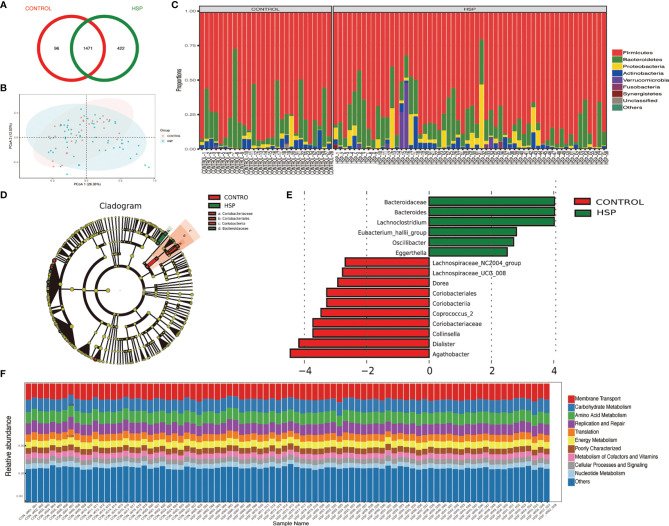
Intestinal dysbiosis between HSP group and control group. **(A)** Venn diagram of OTU distribution in HSP group and control group. **(B)** PCoA analysis between HSP group and control group. **(C)** Histogram of the distribution of intestinal flora in the HSP group and control group at the phylum level. **(D)** LDA evolutionary branch diagram by LEfSe analysis. **(E)** Intestinal flora LEfSe analysis diagram (LDA >2, p < 0.05). **(F)** KEGG function prediction of intestinal microbes in two groups.

A total of 2,159 OTUs are reserved for subsequent analysis and species distribution. The Venn diagram shows that there are 1,471 OTUs coexisting in the HSP group and the control group, accounting for 77.71 and 93.87% respectively (**Figure 1A**). Compared with the CON group, there were no significant differences in microbial diversity (Shannon index p = 0.731, Simpson index p = 0.991) and richness (Chao index, p = 0.349) in the HSP group, which shows that there is no significant difference in the abundance and diversity of intestinal flora between the HSP group and the control group. The structure of the tract flora between HSP group and CON group is similar in the PCOA analysis (**Figure 1B**). At the phylum level, a total of 15 phyla were detected in the two groups (**Figure 1C**). Among them, *Firmicutes*, *Bacteroidetes*, *Proteobacteria*, *Actinobacteria*, *Verrucomicrobia*, *Fusobacteria*, and *Synergistetes* are the seven major abundant.

Sixteen kinds of special gut flora were detected differences between the two groups by LEfSe analysis (**Figures 1D, E**; **Supplementary Table 1**). In the HSP group, *Bacteroidaceae* plays a dominant role, while *Coriobacteriaceae* plays an important role in the healthy control group from class to family level. At the genus level, 12 different floras were found. The main different bacterial genera of HSP include *Bacteroides*, *Lachnoclostridium* and *Eubacterium-hallii group, Oscillibacter*, *Eggerthella*; and in the control group, *Collinsella*, *Agathobacter*, *Dorea*, *Coprococcus-2*, *Lachnospiracea_NC2004 group*, and *UCG-008 group*, *dialister* are dominant. Although the two groups have differential flora, in terms of the function of intestinal microbes, there is no significant difference between the two groups by the KEGG function prediction (**Figure 1F**).

### 3.3 Differential Intestinal Flora Among the Subgroups of HSP

**Figure 2:**
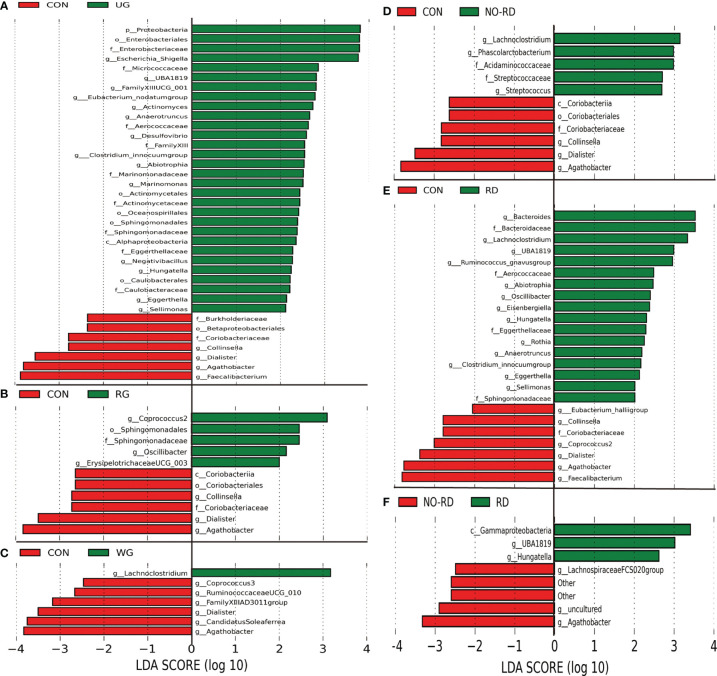
Differences in intestinal flora among the subgroups of HSP and healthy group. **(A)** Intestinal flora LEfSe analysis diagram between the untreated group (UG) and control group (CON). **(B)** Intestinal flora LEfSe analysis diagram between the regular treated group (RG) and control group (CON). **(C)** Intestinal flora LEfSe analysis diagram between the withdrawal group (WG) and control group (CON). **(D)** Intestinal flora LEfSe analysis diagram between the no renal damage group (NO-RD) and control group (CON). **(E)** Intestinal flora LEfSe analysis diagram between the renal damage group (RD) and control group (CON). **(F)** Intestinal flora LEfSe analysis diagram between the no renal damage group (NO-RD) and renal damage group (RD). All LDA >2, p < 0.05.

By using Anosim analysis based on the Bray–Curtis algorithm, it was found that the community structure of the UG and RD group compared separately with healthy children was significantly different (p <0.05), while between the other subgroups did not show this difference. There are also some differential gut floras between the 5 subgroups of HSP with the healthy group by LEfSe analysis (LDA >2, p <0.05) (**Figures 2A–E**). In **Figure 2F**, seven kinds of gut microbiota were found to be significantly different between the NO-RD group and the RD group. However, there was no differential gut flora were found among the UG group, RG group, and WG group.

### 3.4 Changes of Intestinal Metabolites

A total of 578 kinds of metabolites were found in the stool samples from HSP children and healthy control groups. Among them, 59 were significantly different between the two groups (p <0.05), lipids and peptides are the majority (**Table 1**). A total of 32 different metabolites were significantly increased compared to the healthy control group (FC >1), such as 11-keto-β-boswellic acid, enoxolone, and most of the peptides, while 27 different metabolites were significantly reduced (FC <1), including most of the PUFAs such as AA. The VIP value of Cyclohexylsulfamate was the highest, indicating that it may have a greater impact on the occurrence of HSP disease. Total ion chromatogram (TIC) chart shows that the response intensity and retention time of each chromatographic peak basically overlap, indicating that the variation caused by instrument error is small during the entire experiment. Additionally, PCA shows that the QC samples in positive and negative ion modes are closely clustered together, indicating that the repeatability of the experiment is also good and the QC sample correlation map by Pearson analysis also showed a good correlation (**Supplementary Figure 1**).

**Table 1:** Significantly different metabolites between HSP group and healthy control group.

class	adduct	Metabolites	VIP	FC	p-value	m/z	rt(s)
Lipids	(M−H)−	13(S)-Hydroxyoctadecadienoic acid	1.807	0.614	0.046	295.228	93.236
Lipids	(M+H−H2O)+	1-Palmitoylglycerol	5.704	0.084	0.004	313.273	63.788
Lipids	(M+H)+	all cis- (6,9,12)-Linolenic acid	1.846	0.509	0	279.231	91.423
Lipids	(M+NH4)+	Alpha-Linolenic acid	1.266	0.303	0	296.265	64.367
Lipids	(M+H)+	Arachidonic Acid	1.685	0.439	0	305.247	89.608
Lipids	(M+CH3COO+2H)+	cis-9-Palmitoleic acid	2.043	0.484	0.001	315.252	157.651
Lipids	(M+H)+/(M−H)−	Daidzein	2.251	0.361	0.012	255.064	89.115
Lipids	(M−H)−	Dodecanoic acid	3.648	0.199	0.025	199.17	93.132
Lipids	(M+H)+	Geranylgeraniol	1.564	0.417	0.005	291.267	68.07
Lipids	(M+NH4)+	Linoleic acid	2.216	0.634	0.025	298.274	84.196
Lipids	(M+H−H2O)+	Oleic acid	1.437	0.374	0.003	265.252	67.42
Lipids	(M+H)+	Eicosapentaenoic acid	6.146	0.408	0.009	303.231	65.731
Lipids	(M+H)+	Formononetin	1.119	4.606	0.048	269.08	75.902
Lipids	(2M+H)+	L-Norleucine	1.715	2.934	0.003	263.196	493.524
Peptides	(2M+H)+	DL-Phenylalanine	1.129	2.019	0.008	331.164	479.924
Peptides	(M+H−H2O)+	Dopamine	1.182	1.423	0.025	136.075	566.657
Peptides	(M−H)−	D-Proline	1.269	1.932	0.033	114.056	596.733
Peptides	(M+H)+/(M−H)−	L-Alanine	1.447	1.496	0.004	90.054	657.032
Peptides	(M+H)+/(M−H)−	L-Glutamine	1.577	1.6	0.006	147.075	710.784
Peptides	(M+H)+/(M−H)−	L-Leucine	6.368	1.55	0.002	132.101	493.523
Peptides	(M+H)+	L-Pyroglutamic acid	1.468	1.483	0.018	130.049	710.159
Peptides	(M+H)+	L-Threonine	1.729	2.082	0.001	120.064	670.104
Peptides	(M+H)+/(M−H)−	L-Tryptophan	2.791	1.879	0.003	205.096	483.159
Peptides	(M+H)+/(M−H)−	L-Tyrosine	2.767	1.437	0.034	182.081	566.562
Peptides	(M+H)+/(M−H)−	L-Valine	3.077	1.491	0.032	118.085	567.527
Peptides	(M+NH4)+	Methoprene (S)	1.864	0.216	0	328.284	64.941
Phenylpropanoids	(M−H)−	Cyclohexylsulfamate	25.894	0.41	0.004	178.054	134.362
Phenylpropanoids	(M+H)+	trans-2-Hydroxycinnamic acid	1.574	1.419	0.027	165.054	566.562
Phenylpropanoids	(M−H)−	trans-cinnamate	1.119	1.409	0.008	147.045	482.794
Nucleic acids	(M−H)−	Thymine	3.144	1.784	0.013	125.035	131.713
Nucleic acids	(M+H−H2O)+	Tyramine	4.687	1.532	0.002	120.08	479.944
Nucleic acids	(M+H)+	Uracil	2.953	1.363	0.04	113.034	162.164
Carbohydrates	(M−H)−	D-Lyxose	2.345	0.602	0	149.045	302.31
Carbohydrates	(M+CH3COO)−	D-Ribose	1.065	0.643	0.004	209.067	303.28
Carbohydrates	(M+H−H2O)+	N-Acetylmannosamine	1.013	2.249	0.002	204.086	204.259
Organic acids	(M−H2O−H)−	2-Oxoadipic acid	3.112	0.588	0.03	141.017	653.781
Organic acids	(M+H)+/(M−H)−	L-Phenylalanine	6.419	1.56	0.002	166.086	479.929
Enzymes	(M−H)−	Enalapril	1.698	3.048	0.029	375.184	335.598
Terpenoids	(M+H)+	Enoxolone	3.645	5.091	0.029	471.346	93.061
Amino acid related compounds	(M+H)+	Capsaicin	1.114	0.319	0.006	306.206	72.841
Vitamins	(M−H)−	Nicotinate	1.389	0.748	0.004	122.025	430.316
Other	(M−H)−	11-Keto-beta-boswellic acid	3.341	11.071	0.024	469.331	93.427
Other	(M+CH3CN+H)+	16-Hydroxypalmitic acid	1.86	0.434	0	314.268	92.142
Other	(M+H)+	1-Palmitoyl-2-hydroxy-sn-glycero-3-phosphoethanolamine	1.764	0.519	0.008	454.291	372.123
Other	(M+H)+	20-Hydroxyarachidonic acid	2.009	0.449	0.004	321.241	66.89
Other	(M−H+2Na)+	2-Butoxyethanol	1.051	0.603	0.047	163.075	75.237
Other	(M+H)+	2-Deoxyinosine	1.732	1.552	0.03	253.093	338.621
Other	(M−H)−	2-hydroxy-butanoic acid	1.338	1.893	0.011	103.04	364.888
Other	(M−H)−	3-(3-Hydroxyphenyl) propanoic acid	4.646	2.124	0.034	165.056	213.446
Other	(M−H)−	3,4-Dihydroxyhydrocinnamic acid	1.291	2.316	0.048	181.051	419.406
Other	(M+H−H2O)+	DL-Indole-3-lactic acid	2.88	1.762	0.003	188.07	482.906
Other	(M+H)+/(M−H)−	DL-Methionine sulfoxide	2.449	2.475	0	166.052	698.598
Other	(M+H)+	Erucamide	1.216	0.254	0.025	338.341	67.109
Other	(M−H)−	Gentisic acid	1.432	2.331	0.039	153.019	135.753
Other	(M+H)+	MG(18:2(9Z,12Z)/0:0/0:0)	3.625	0.398	0	355.283	69.542
Other	(M−H)−	N-Acetyl-L-aspartic acid	1.136	0.462	0.001	174.041	773.394
Other	(M+H−H2O)+	Phenyllactic acid	1.026	1.511	0.002	149.059	479.928
Other	(M+H)+	Phe-Pro	1.181	1.369	0.039	263.138	445.378
Other	(M−H)−	Traumatic Acid	1.1	0.298	0.028	227.129	576.333

FC, Fold change; m/z, mass-to-charge ratio; rt, retention time.

**Figure 3:**
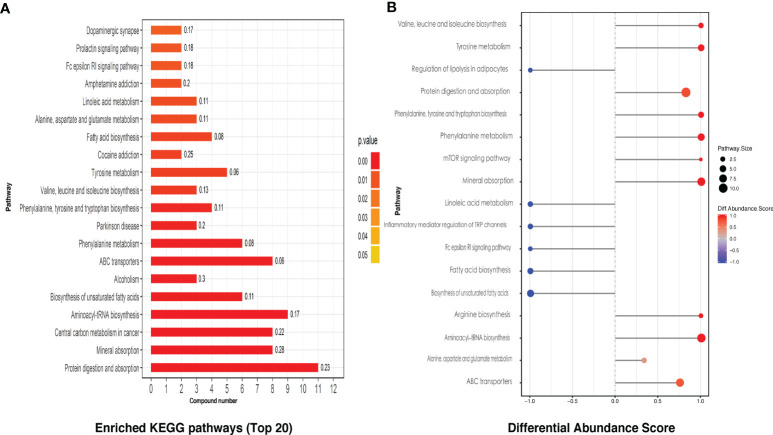
The KEGG pathway analysis of intestinal metabolites. **(A)** The differential metabolites related top 20 of the enriched KEGG pathways. **(B)** Differential intestinal metabolites abundance analysis of KEGG pathways.

KEGG pathway analysis screened a total of 28 enriched metabolic pathways. The top 20 KEGG pathways are shown in **Figure 3A**, which mainly include protein digestion and absorption, mineral absorption, unsaturated fatty acid biosynthesis, and partial amino acid biosynthesis and metabolism, linoleic acid metabolism, etc., involving the immune, nervous system, amino acid, and lipid metabolism, digestion, signal transduction, and other secondary levels. By differential abundance analysis, we can see interestingly that four kinds of fatty acid related KEGG pathways were all displayed a negative differential abundance score (**Figure 3B**).

### 3.5 Differences in Intestinal Metabolites Among the Subgroups of HSP and Healthy Group

**Figure 4:**
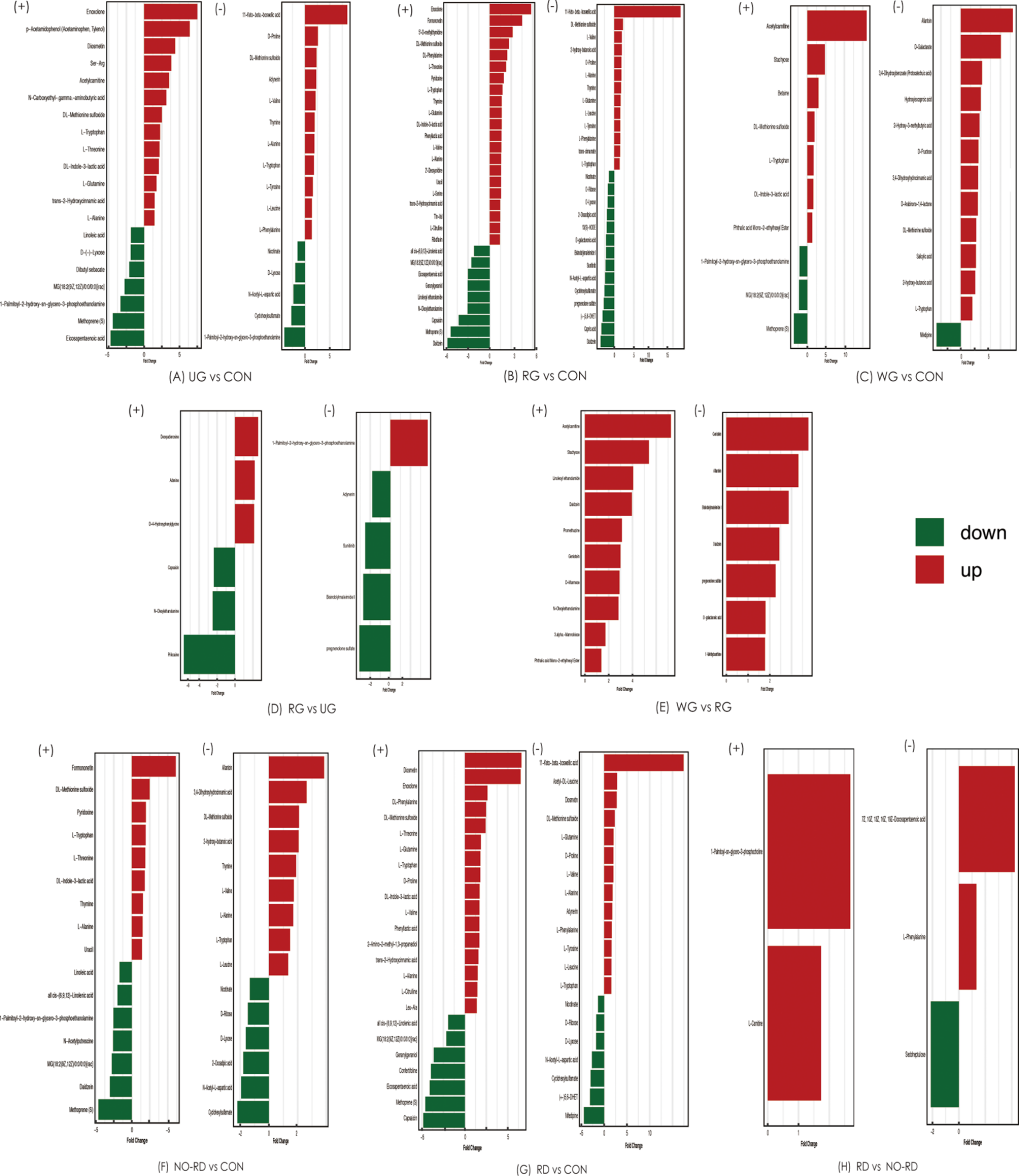
Differential gut metabolites among 5 subgroups of HSP and healthy groups in both positive and negative ion modes. (+) shows positive ions, and (−) shows negative ions. UG, the untreated group; CON, the healthy control group; RG, the regular treated group; WG, the withdrawal group; NO-RD, no renal damage group, RD: renal damage group.

Comparison analysis of metabolites was tested among five subgroups of HSP and healthy groups. These differential metabolites are shown in **Figure 4**. In general, compared with CON groups, lipids, and peptides in five subgroups of HSP also were the majority, while between the subgroups, the differential gut metabolites were less and no disease-related differences were found.

### 3.6 Correlation Analysis Between Differential Gut Microbiota and Metabolites

**Figure 5:**
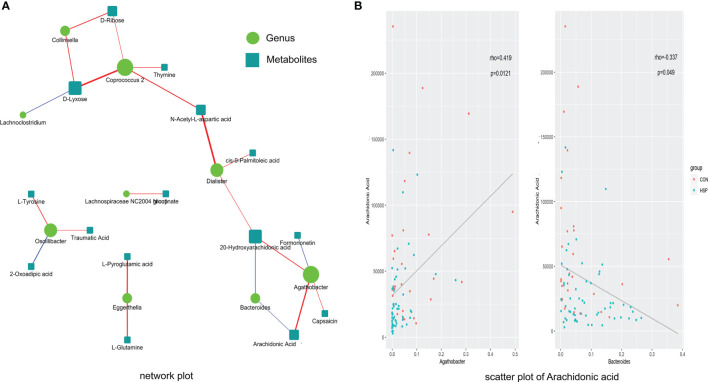
Spearman correlation between significantly different intestinal microbes and metabolites. **(A)** Spearman correlation analysis network diagram of significant difference flora and significant metabolites. Red lines mean positive correlation and blue lines mean negative correlation. The darker the color, the stronger the correlation. **(B)** Scatter plot of AA with *Agathobacter* (left) *and Bacteroides* (right).

The abundance of 12 different bacterial genera (LEfSe LDA >2 and p <0.05) and 59 significantly different metabolites (VIP >1 and p <0.05) at the above-mentioned were subjected to Spearman correlation analysis to calculate their correlation coefficients. A total of 22 pairs of significantly related different bacterial genera and metabolites (|r| >0.3 and p <0.05) were found, and 17 pairs of flora-metabolites with significant positive correlation, and 5 pairs of flora-metabolites with significant negative correlation (**Figure 5A** and **Supplementary Table 2**). Among them, *Dialister* and N-acetyl-L-aspartic acid (r = 0.46, p <0.01), *Coprococcus-2* and D-xylose were significantly positively correlated (r = 0.44, p <0.01). Moreover, the fatty acid metabolites we are concerned about decreased AA in HSP are negative closely related to *Bacteroides* and positive to *Agathobacter* (**Figure 5B**). As mentioned in **Figure 1E** before, *Bacteroides* were significantly increased and *Agathobacter* was significantly decreased in the HSP group, which implies AA may participate in the pathogenesis of HSP.

### 3.7 Serum AA and its Metabolites Analysis

**Figure 6:**
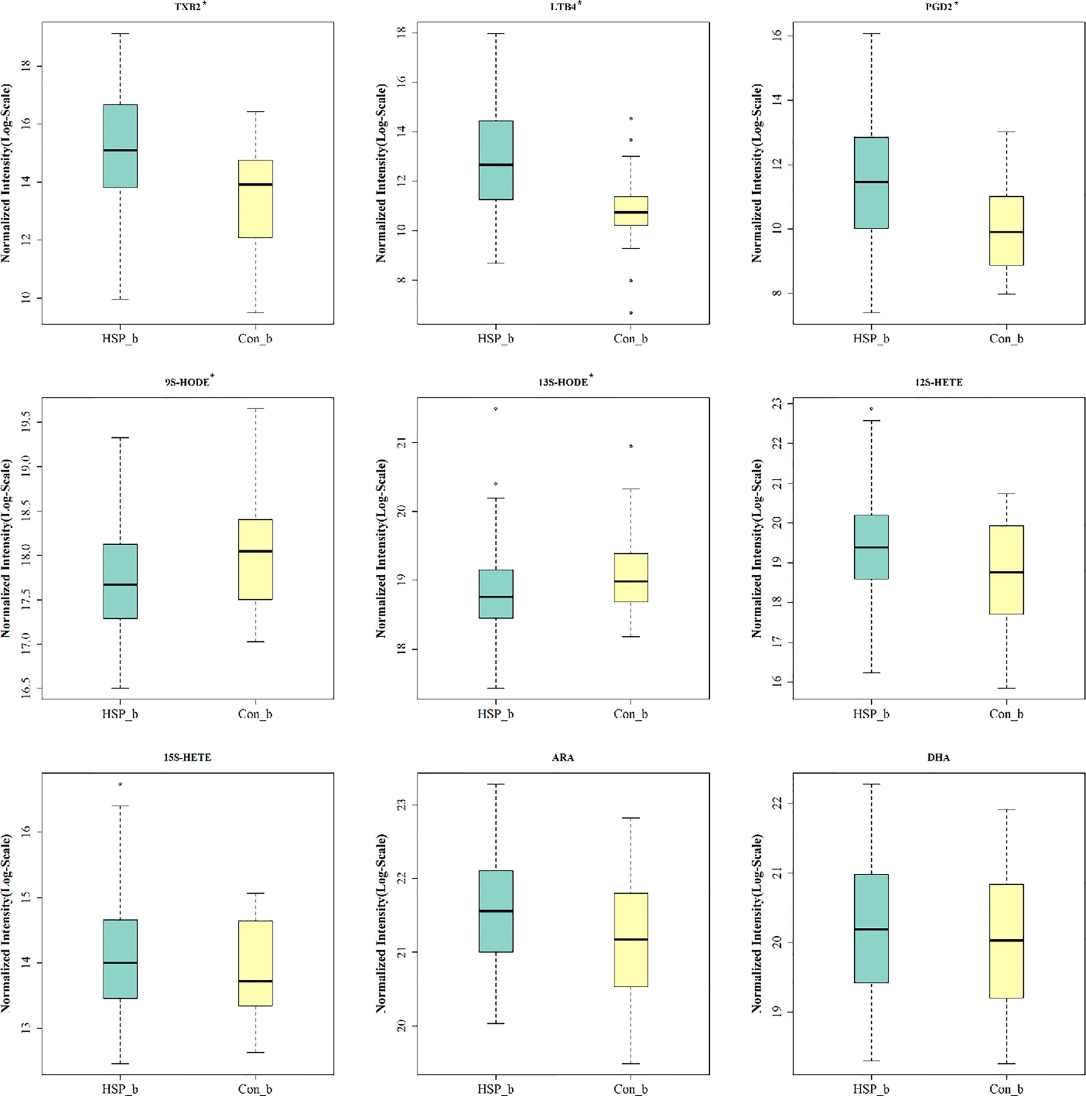
Serum AA and its metabolites analysis. * means p < 0.05.

Common 15 kinds of AA and its metabolites were tested by MRM. A total of nine metabolites were detected in the serum of two groups (**Figure 6**). Among them, five metabolites are significantly different, which are elevated TXB2, LTB4, PGD2, and also reduced 9S-HODE, and 13S-HODE. The AA content in HSP groups was higher than that in the CON group, but there was no significant difference.

## 4 Discussion

Our study delivered an integrated analysis of gut microbiome and host metabolome in children with HSP. HSP group had a dominant *Bacteroidaceae* and decreased *Coriobacteriaceae* in the family level of gut microbes. A total of 59 kinds of gut metabolites significantly differ between the two groups, in which most are lipids and peptides. AA-related metabolites TXB2, LTB4, PGD2, 9S-HODE, and 13S-HODE were significantly changed. In conclusion, the results of this investigation suggested that the occurrence and development of HSP may be related to the biosynthesis and metabolism of unsaturated fatty acids especially AA and its metabolites through gut microbiota and metabolome analysis.

The intestinal microecosystem is the most important and complex in the human body. The occurrence of many diseases such as HSP is closely related to the disorder of gut microbiota. In 2018, a study on intestinal flora of children with HSP (Wang et al., 2018) revealed that the abundance of *Fusobacteria* in the HSP group was higher than that in the healthy control group at the phylum level, while the abundance of *Firmicutes* was lower. At the genus level, the abundance of *Dialister* and *Roseburia* in the HSP group was lower than that in the control group, while *Parabacteroides* and *Enterococcu*s were higher. During the same period, the center delivered another research on oral mucosa microorganisms in children with HSP (Chen et al., 2018) found that *Bacteroides* were dominant in the HSP group, while *Proteobacteria* was dominant in the healthy control group. Consistent with previous studies, our results of 16S rRNA showed *Bacteroides* was dominant in children with HSP, while the abundance of *Dialister* was decreased in all subgroups. *Bacteroides* is a gram-negative anaerobic Bacillus sp., which is common in human and animal intestines, oral cavity, and reproductive tract. It can promote and regulate the differentiation of T cells, inhibit the inflammatory reaction and other functions, and has an impact on the production of SCFAs (Chen et al., 2017; Wexler and Goodman, 2017). *Dialister*, belonging to *Firmicutes*, is an anaerobic acinetobacter, which is closely related to the production of fatty acids (Afouda et al., 2020). A study on intestinal microflora in infants with food allergy (Savage et al., 2018) found that the colony counts of *Dialister* and *Dorea* in children with allergic symptoms were lower than those in the control group, indicating that the two floras were significantly related to food allergy. In addition, a study on the intestinal flora of juvenile idiopathic arthritis (Qian et al., 2020) also showed that the abundance of *Dialister* was lower than that of the normal control group, which may be related to the immunomodulatory effect of fatty acids. *Agathobacter* species are anaerobic, gram-positive bacteria in the family Lachnospiraceae. The main fermentation products are butyrate, acetate, hydrogen, and lactate (Hua et al., 2020). *Agathobacter* was found to be significantly dominant not only in healthy control groups compared with all subgroups but also in the NO-RD group compared with the RD group. Therefore, it seems that *Agathobacter* may be a kind of universal bacteria in healthy people and the decrease of it may have some links to the renal damage in HSP. However, there was no differential gut flora among the UG group, RG group, and WG group, which might be limited to the small samples of each subgroup.

Fatty acids were important differential metabolites in the HSP group. AA, oleic acid, Eicosapentaenoic acid (EPA), linolenic acid, and linoleic acid were all detected to be reduced in HSP groups (de Carvalho and Caramujo, 2018). According to the KEGG enrichment pathway, the biosynthesis of fatty acids, especially unsaturated fatty acids, was importantly affected. Saturated fatty acids such as palmitic acid can stimulate proximal renal tubular epithelial cells to release extracellular vesicles, which can promote the occurrence of renal insufficiency based on metabolic diseases (Cobbs et al., 2019). Monounsaturated fatty acids such as oleic acid have the ability to regulate cholesterol metabolism, and long-chain monounsaturated fatty acids have been shown to reduce the development of atherosclerosis in mouse models (Yang et al., 2017). AA is a kind of omega-6 polyunsaturated fatty acid, which is mainly expressed as a phospholipid in the cell membrane and released from phospholipid under the action of phospholipase A2 and phospholipase C (Wang et al., 2019). It has a series of physiological activities, such as esterifying cholesterol, increasing vascular elasticity, reducing blood viscosity, and regulating blood cell function. It can synthesize bioactive substances, such as prostaglandins, Thromboxane, and leukotriene play a very important role in the human immune system (Collu et al., 2020). EPA has an anti-atherosclerotic effect and has a significant effect on reducing blood lipid and inhibiting platelet aggregation (de Carvalho and Caramujo, 2018). Surprisingly, the results showed that the organic acid metabolites such as linolenic acid, linoleic acid, AA, EPA, and oleic acid were lower than those in the healthy control group, and they all participated in the key part of the biosynthesis pathway of unsaturated fatty acids. To some extent, the onset of HSP may inhibit the biosynthesis of fatty acids, especially unsaturated fatty acids.

In this study, Spearman correlation analysis was conducted on the intestinal flora and metabolites with significant differences in HSP. The results showed that there were 22 pairs of significantly related differential flora and metabolites. Among them, *Dialister* and N-acetyl-L-aspartic acid (r = 0.46, p <0.01) were significantly correlated. Association analysis revealed that *Coprococcus-2* and *Agathobacter* were related to the changes of various metabolites, suggesting that these two groups may be an entry point for further study. Recent researches have examined that unsaturated fatty acids reduced may have a stimulating effect on the growth of *Bacteroides* (Robertson et al., 2018). Wang et al. (Tan et al., 2021) found AA had a positive correlation with *Bacteroides* in the disease of chronic urticaria. On the contrary, in our results, downregulated AA in HSP is negative closely related to *Bacteroides* and positive to *Agathobacter*, which explains that the relationship between AA and *Bacteroides* is related but not limited to a positive regulatory relationship. The links between AA and *Agathobacter* have not been discovered before. Due to this, it provides a new sight of exploring the positive correlation between AA and *Agathobacter* in future studies.

Another important finding was that five kinds of AA related metabolites were found to be significantly increased in the serum samples of HSP children, mainly related to the COX pathway and LOX pathway (Wang et al., 2019). Early in 1986, a study found that abnormalities of vascular prostaglandin metabolism may be involved in the pathophysiology of HSP (Turi et al., 1986). Another study tested the blood and urinary TLB4 in patients with HSP and suggested that Leukotrienes may play a proinflammatory and profibrotic role in the pathogenesis of HSP (Wu et al., 2009). Demir et al. (2020) made the conclusion that DHAP (18:0), PGD2/I2, porphobilinogen, 5-methyltetrahydrofolic acid, and N-Acetyl-4-O-acetylneuraminic acid/N-Acetyl-7-O-acetylneuraminic acid may serve as biomarkers for predicting HSP with kidney disease through an untargeted metabolomics approach in serum. 9S- and 13S-HODEs, oxidized linoleic acid metabolites and derived from linoleic acid, can reflect the status of oxidative stress and are linked to inflammation, atherogenesis, metabolic syndrome, and stroke (Vangaveti et al., 2016; Kotlega et al., 2021). 9S/13S HODE were declined in a study of stroke, and at last, indicating the most important inflammatory derivatives of free fatty acids related to cognitive decline after stroke (Kotlega et al., 2021). Therefore, it is speculated that the AA metabolites may be related to the pathogenesis of HSP by the COX pathway and LOX pathway.

The research had a major limitation due to the limited sample size and potential errors during sample collection, needing more research to verify. Although the interaction between intestinal microbiota and host metabolites in the disease of HSP is still unclear and sometimes over-explained (Rastelli et al., 2019), some intestinal microbiota and metabolites especially unsaturated fatty acids that can affect host physiology will be discovered in the future. Our results may provide potential biomarkers for the diagnosis of HSP and could be a guide for the treatment.

## Data Availability Statement

The data presented in the study are deposited in the figshare repository, accession number with DOI: 10.6084/m9.figshare.16715389.

## Ethics Statement

The studies involving human participants were reviewed and approved by the Ethics Committee of the Second Xiangya Hospital of Central South University (No. XXJ2018-11). Written informed consent to participate in this study was provided by the participants’ legal guardian/next of kin.

## Author Contributions

HXJ designed the experiment and provided guidance and support in the whole process. MW, DXQ, LXY and LTH participated in the experiments, data analysis, and manuscript writing. HQN and FSP are responsible for the specimen collection and technical guide. All authors contributed to the article and approved the submitted version.

## Funding

This work was supported by the Fundamental Research Funds for the Central Universities of Central South University under Grant [number 2019zzts811].

## Conflict of Interest

The authors declare that the research was conducted in the absence of any commercial or financial relationships that could be construed as a potential conflict of interest.

## Publisher’s Note

All claims expressed in this article are solely those of the authors and do not necessarily represent those of their affiliated organizations, or those of the publisher, the editors and the reviewers. Any product that may be evaluated in this article, or claim that may be made by its manufacturer, is not guaranteed or endorsed by the publisher.
