# Supplementary material for: Integrated Analyses of Gut Microbiome and Host Metabolome in Children With Henoch-Schönlein Purpura
Source: Front Cell Infect Microbiol. 2022 Jan 25;11:796410. doi: 10.3389/fcimb.2021.796410 (PMC8821812; doi:10.3389/fcimb.2021.796410)
Supplement: Supplementary file 1 [file DataSheet_1.docx]

| Flora name | logarithm value | groups | LDA value | p value | level |
| --- | --- | --- | --- | --- | --- |
| *Eubacterium_hallii_group* | 3.465 | HSP | 2.796 | 0.020 | Genus |
| *Bacteroides* | 4.926 | HSP | 4.022 | 0.035 | Genus |
| *Oscillibacter* | 3.228 | HSP | 2.707 | 0.041 | Genus |
| *Bacteroidaceae* | 4.926 | HSP | 4.022 | 0.035 | Family |
| *Lachnoclostridium* | 4.566 | HSP | 4.011 | 0.018 | Genus |
| *Eggerthella* | 2.885 | HSP | 2.504 | 0.008 | Genus |
| *Agathobacter* | 4.932 | CONTROL | 4.453 | 0.001 | Genus |
| *Dorea* | 3.792 | CONTROL | 2.925 | 0.030 | Genus |
| *Coprococcus_2* | 4.047 | CONTROL | 3.465 | 0.016 | Genus |
| *Coriobacteriia* | 4.022 | CONTROL | 3.282 | 0.023 | Class |
| *Dialister* | 4.603 | CONTROL | 4.179 | 0.000 | Genus |
| *Lachnospiraceae_NC2004_group* | 2.246 | CONTROL | 2.689 | 0.002 | Genus |
| *Coriobacteriaceae* | 3.951 | CONTROL | 3.724 | 0.001 | Family |
| *Collinsella* | 3.951 | CONTROL | 3.724 | 0.001 | Genus |
| *Coriobacteriales* | 4.022 | CONTROL | 3.282 | 0.023 | Order |
| *Lachnospiraceae_UCG_008* | 1.772 | CONTROL | 2.775 | 0.023 | Genus |

**Supplementary Table 1.** Differences in species between HSP group and healthy control group


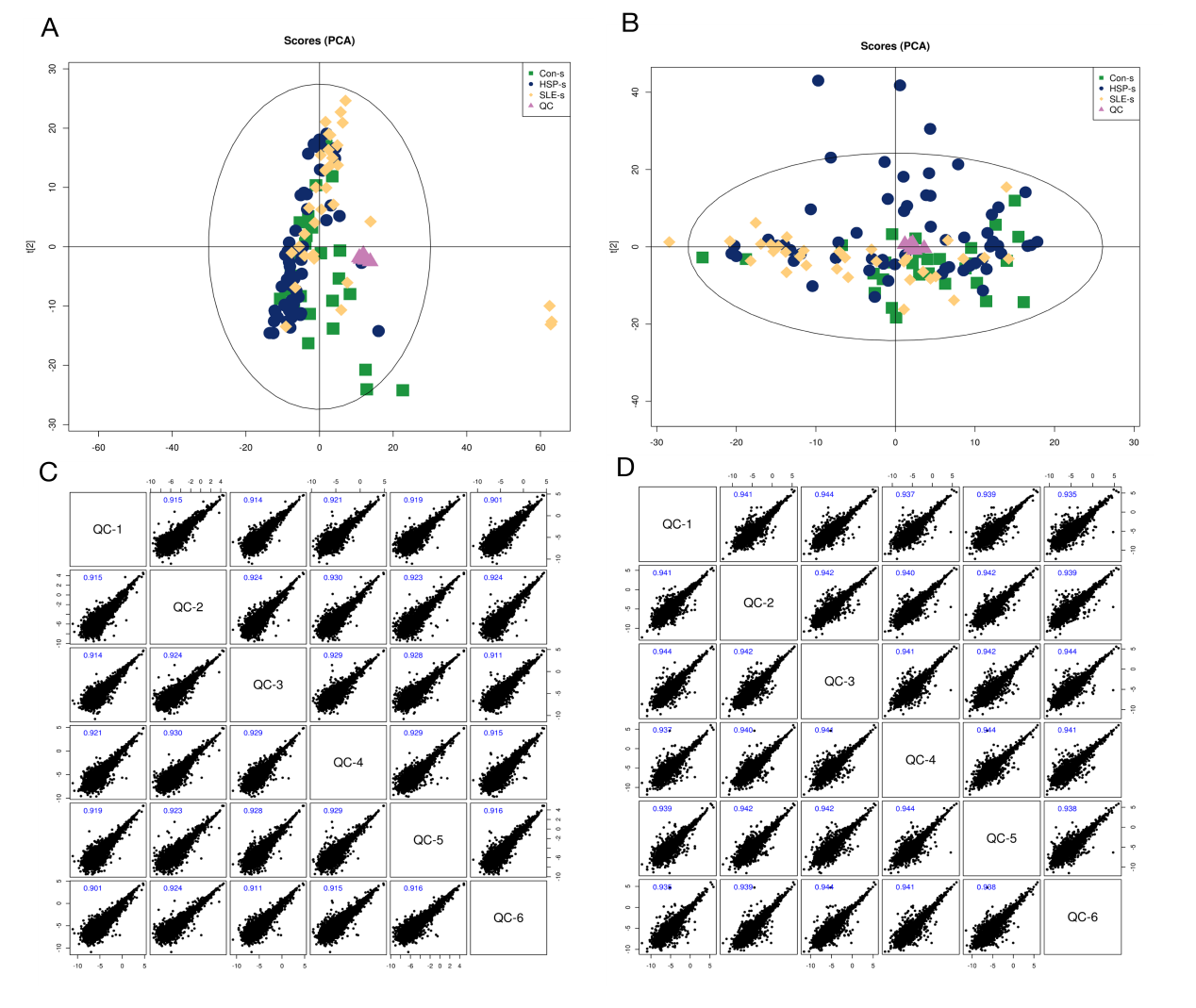


**Supplementary Figure 1.** PCA score graph and QC sample correlation map in positive and negative ion mode. A: PCA score graph in positive ion mode (parameters R2X=0.152cum, R2Y=0.885cum, Q2=0.414cum). B: PCA score graph in negative ion mode (parameters R2X=0.0985cum, R2Y=0.738cum, Q2=0.174cum). C: QC sample correlation map in positive ion mode. D: QC sample correlation map in negative ion mode.

**Supplementary Table 2.** Correlation analysis between differential gut microbiota and metabolites

| Metabolites | Genus | Coefficient | p value | ABS | LABLE |
| --- | --- | --- | --- | --- | --- |
| N-Acetyl-L-aspartic acid | Dialister | 0.461 | 0.006 | 0.461 | pos |
| D-Lyxose | Coprococcus 2 | 0.444 | 0.007 | 0.444 | pos |
| Arachidonic Acid | Agathobacter | 0.419 | 0.012 | 0.419 | pos |
| L-Pyroglutamic acid | Eggerthella | 0.416 | 0.012 | 0.416 | pos |
| L-Glutamine | Eggerthella | 0.404 | 0.016 | 0.404 | pos |
| 2-Oxoadipic acid | Oscillibacter | -0.380 | 0.029 | 0.380 | neg |
| 20-Hydroxyarachidonic acid | Agathobacter | 0.372 | 0.029 | 0.372 | pos |
| D-Lyxose | Collinsella | 0.374 | 0.029 | 0.374 | pos |
| D-Ribose | Collinsella | 0.377 | 0.029 | 0.377 | pos |
| L-Tyrosine | Oscillibacter | 0.370 | 0.029 | 0.370 | pos |
| N-Acetyl-L-aspartic acid | Coprococcus 2 | 0.384 | 0.029 | 0.384 | pos |
| 20-Hydroxyarachidonic acid | Bacteroides | -0.359 | 0.041 | 0.359 | neg |
| D-Lyxose | Lachnoclostridium | -0.355 | 0.043 | 0.355 | neg |
| Formononetin | Agathobacter | -0.353 | 0.044 | 0.353 | neg |
| Nicotinate | Lachnospiraceae NC2004 group | 0.350 | 0.044 | 0.350 | pos |
| 20-Hydroxyarachidonic acid | Dialister | 0.340 | 0.048 | 0.340 | pos |
| Capsaicin | Agathobacter | 0.339 | 0.048 | 0.339 | pos |
| cis-9-Palmitoleic acid | Dialister | 0.342 | 0.048 | 0.342 | pos |
| D-Ribose | Coprococcus 2 | 0.339 | 0.048 | 0.339 | pos |
| Thymine | Coprococcus 2 | 0.339 | 0.048 | 0.339 | pos |
| Traumatic Acid | Oscillibacter | 0.341 | 0.048 | 0.341 | pos |
| Arachidonic Acid | Bacteroides | -0.337 | 0.049 | 0.337 | neg |
